# Alterations and Dynamics of Major Meningitis Etiological Agents During and Post-COVID-19 Pandemic: A Systematic Review

**DOI:** 10.3390/tropicalmed10030081

**Published:** 2025-03-18

**Authors:** Luís Arthur Brasil Gadelha Farias, Larissa Santos Weyne, Lenifer Siqueira Landim, Pablo Eliack Linhares de Holanda, Aliniana da Silva Santos, Luciano Pamplona de Góes Cavalcanti, Lourrany Borges Costa, Antonio Gutierry Neves Dantas de Melo, Melissa Soares Medeiros, Evelyne Santana Girão, Tânia Mara Silva Coelho, Lauro Vieira Perdigão Neto

**Affiliations:** 1 Laboratório de Investigação Médica—LIM 49, Department of Infectious Diseases of Hospital das Clínicas, University of São Paulo, São Paulo 05508-220, Brazil; lauro_perdigao@hotmail.com; 2 São José Hospital for Infectious Diseases, Fortaleza 60455-610, Brazil; eliack2@hotmail.com; 3 Christus University Center (Unichristus), Fortaleza 60160-230, Brazil; lsantosweyne@gmail.com (L.S.W.); lesiqueirauni@gmail.com (L.S.L.); aliniana.ssantos@gmail.com (A.d.S.S.); pamplona.luciano@gmail.com (L.P.d.G.C.); melmedeiros@hotmail.com (M.S.M.); coelhotaniamara@gmail.com (T.M.S.C.); 4 Public Health Department, Medicine Faculty, Federal University of Ceará, Fortaleza 60355-636, Brazil; lourranybc@gmail.com; 5 Public Health Department, Medicine Faculty, Fortaleza University (Unifor), Fortaleza 60811-905, Brazil; 6 Medicine Faculty, Federal University of Ceará, Fortaleza 60355-636, Brazil; gutierrydemelo@alu.ufc.br (A.G.N.D.d.M.); egirao@uol.com.br (E.S.G.)

**Keywords:** COVID-19, meningitis, enterovirus, *Haemophilus influenzae*, *Neisseria meningitidis*, pandemic, *Streptococcus pneumoniae*

## Abstract

The transmission dynamics of many pathogens were altered during the coronavirus disease 2019 (COVID-19) pandemic. Several factors, including control measures and social distancing, have influenced the circulation and epidemiology of major etiological agents of meningitis during this period. This review examined trends in the primary etiologic agents of meningitis during and after the COVID-19 pandemic. A comprehensive literature search was conducted using the MEDLINE, Embase, LILACS, and SciELO databases for studies published between 2020 and 2024. The data were summarized descriptively and reported according to the Preferred Reporting Items for Systematic Reviews and Meta-Analyses guidelines. Thirty-eight studies are included in this review. Bacterial and viral meningitis pathogens exhibited significant epidemiological shifts during the pandemic. A marked decline in infections caused by the enteroviruses, *Neisseria meningitidis*, *Streptococcus pneumoniae*, and *Haemophilus influenzae* was observed from 2020 to 2021 in the northern and southern hemispheres during the pandemic. Post-pandemic, meningitis cases increased, with a resurgence in various countries. Despite the heterogeneity of the studies, the evidence indicates that the COVID-19 pandemic significantly affected the epidemiology of meningitis-causing microorganisms during and after the pandemic. Understanding these epidemiological shifts and dynamics is crucial for defining the control measures, vaccination strategies, and public health policies in the post-COVID-19 era.

## 1. Introduction

Meningitis is a medical condition characterized by the inflammation of the membranes covering the brain and spinal cord. This inflammation can be categorized as acute or chronic, each with a distinct clinical course. Meningitis is usually a medical emergency requiring early diagnosis and treatment to prevent adverse outcomes. Currently, cerebrospinal fluid (CSF) culture remains the gold standard for diagnostic confirmation. However, delays in sample processing prior to antimicrobial therapy initiation can lead to false negative results [1,2]. Advances in laboratory techniques, such as real-time polymerase chain reaction (RT-PCR) panels, have improved the number of accurate diagnoses and reduced the reliance on CSF cultures. In middle- and high-income countries, the widespread use of increasingly sensitive multiplex PCR assays, syndromic testing, and national guideline recommendations have significantly increased the detection rates of bacterial and viral pathogens in CSF [3].

Bacteria and viruses are the primary etiological agents of meningitis [1,2,3]. Recent meta-analyses on the global etiology of meningitis have identified *Neisseria meningitidis* and *Streptococcus pneumoniae* as the main pathogens worldwide. Other important pathogens include *Haemophilus influenzae*, *S. agalactiae*, *Escherichia coli*, and *Listeria monocytogenes* [2]. The etiology of meningitis may vary according to the age group, with *S. agalactiae* being the most common cause in neonates under days [1,2]. *Streptococcus pneumoniae* infection was the most common cause of bacterial meningitis in Europe, Africa, and the Western Pacific, particularly in children [2]. Enteroviruses including coxsackieviruses and echoviruses were the primary causative agents of viral meningitis [1]. Enteroviral infections occur year-round but are most commonly seen in summer and autumn [1]. Other causes include herpesviruses, adenoviruses, and arboviruses, depending on the local epidemiology [1].

During the coronavirus disease (COVID-19) pandemic, global efforts were primarily focused on controlling SARS-CoV-2 transmission and addressing its clinical consequences. The pandemic also altered the circulation of other infectious diseases, mainly respiratory viruses, leading to a decline in influenza, parainfluenza, and adenovirus infections [4]. Recent studies have shown that other agents, such as bacteria, also exhibited changes in their transmission dynamics, which could be related to increased respiratory and sanitary protection measures implemented during the pandemic [5]. Since the beginning of the pandemic, several studies have investigated the circulation of bacterial and viral agents in both acute and chronic meningitis. However, the full extent of the pandemic’s impact on meningitis epidemiology remains unclear [5,6].

Several hypotheses suggest that many factors contributed to the reduced circulation of other etiological agents of meningitis. Lockdown measures, social distancing, and mask use may have played a role in decreasing diseases transmitted through droplets, such as bacterial meningitis [5,6]. Additionally, school closures, reduced international travel, and limited social interactions likely disrupted the transmission dynamics of meningitis pathogens [4,5,6]. Changes in healthcare-seeking behavior, with fewer medical visits during the pandemic may have also influenced disease detection and reporting [5,6]. These control measures, while effective in reducing respiratory infections, may have indirectly impacted the spread of bacterial and viral meningitis, altering their epidemiological patterns during and after the pandemic—a phenomenon that still requires further study.

To the best of our knowledge, few large-scale studies have examined the trends in pathogens responsible for central nervous system infections during and after the COVID-19 pandemic [5,6]. Moreover, most studies were limited to single-center investigations or focused on specific populations and pathogens. Understanding the epidemiological patterns of these pathogens and the factors influencing their circulation during the pandemic can provide valuable insights for future prevention strategies and public health measures. Therefore, we conducted a systematic review to analyze and better understand the trends in viral and bacterial meningitis during and after the COVID-19 pandemic and assess its impact on the circulation of the main etiological agents of meningitis worldwide.

## 2. Materials and Methods

The primary question guiding this review was “has the COVID-19 pandemic influenced the global circulation of meningitis etiological agents?” To address this question, a comprehensive literature search was conducted using the following primary databases: MEDLINE^®^ (via PubMed), Embase, LILACS, and SciELO. The search was from January 2020 to December 2024 and included the following terms to retrieve relevant articles: (“Meningitis” OR “Etiological agents of meningitis” OR “*Streptococcus pneumoniae*” OR “Enterovirus” OR “*Neisseria meningitidis*” OR “*Haemophilus influenzae*” OR “*Streptococcus agalactiae*” OR “*Listeria monocytogenes*”) AND (“COVID-19 Pandemic” OR “COVID-19” OR “New coronavirus” OR “Pandemic.”). This systematic review adhered to the Preferred Reporting Items for Systematic Reviews and Meta-Analyses (PRISMA) guidelines (http://www.prisma-statement.org (accessed on 25 January 2024)). This systematic review was registered on the International Prospective Register of Systematic Reviews (PROSPERO) with registration number CRD420250643445, and the search strategy is detailed in the Supplementary Materials (File S1).

We only included and thoroughly analyzed articles in English, Spanish, and Portuguese. We also reviewed the reference lists of retrieved articles to identify additional relevant studies that might have been missed in the initial search strategy. We included randomized and non-randomized controlled trials, cohort, case–control, cross-sectional, outbreak reports, genomic studies, and systematic literature reviews that included a meta-analysis. However, case reports and series were excluded. A case series was defined as one comprising 20 or fewer patients and characterized by the uncontrolled nature of these studies [7]. Mendeley 2.118.0 (https://www.mendeley.com), a free reference management software, was used to organize references, manage duplicates, and streamline article selection. Full-text articles identified during the screening process were retrieved for detailed analysis.

Studies were included in the review if they reported the following primary outcomes: (1) meningitis epidemiological data, such as incidence, prevalence, or serotype distribution during or post-COVID-19; (2) etiological agents of meningitis, including invasive/atypical disease presentations to better describe trends in pathogen circulation during and post COVID-19; and (3) comparative data on meningitis etiological agents before, during, and after the COVID-19 pandemic. Additionally, studies that described the etiological agents of meningitis across all age groups were considered.

Two independent reviewers assessed the titles and abstracts of articles to select those that met the predefined inclusion criteria. Any discrepancies were resolved by consensus or, if needed, by consulting a third reviewer. Two infectious disease specialists and an epidemiologist with experience in infectious disease epidemiology reviewed the articles. We designed this process to reduce selection bias and ensure comprehensive literature representation. The quality of individual articles was assessed based on the application of standardized checklists for each selected article. Two independent reviewers evaluated this study’s risk of bias and quality using: (1) the Newcastle–Ottawa Scale for observational studies, including cohort and case–control studies; (2) the Joanna Briggs Institute (JBI) Critical Appraisal Checklist for analytical cross-sectional studies; and (3) a Measurement Tool to Assess Systematic Reviews 2 (AMSTAR-2) for systematic reviews and meta-analysis studies.

The data were categorized by etiological agent whenever available and were further classified into bacterial or viral meningitis when the specific pathogen was not identified or specified in the article. For this review, the pre-pandemic period was defined as 2019, the pandemic period spanned from January 2020 to December 2021, and the post-pandemic period encompassed 2022 and subsequent years.

## 3. Results

### 3.1. Study Selection Method

**Figure 1:**
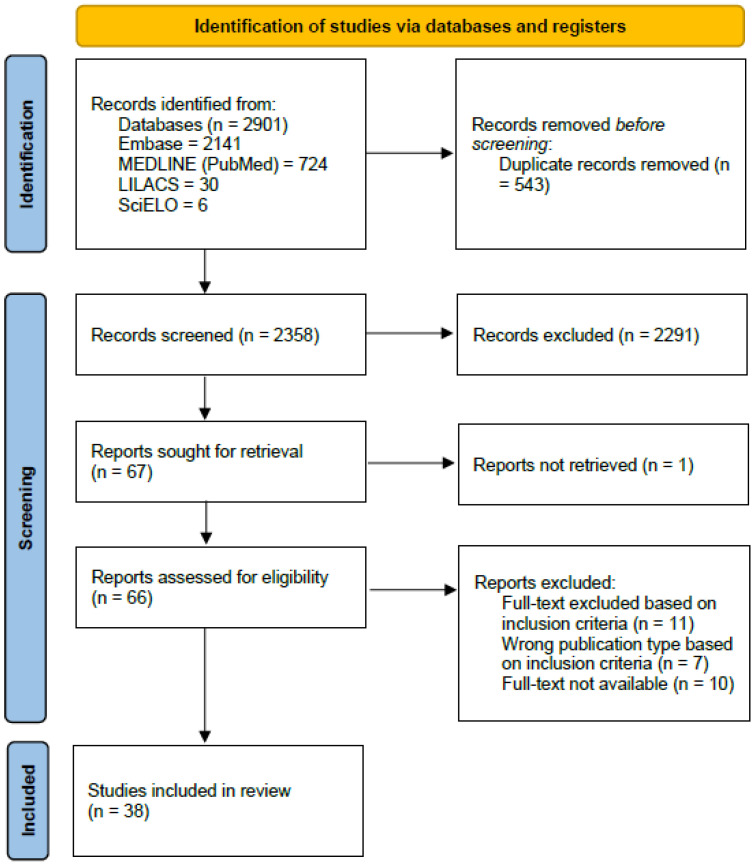
PRISMA flow diagram showing a schematic illustration of database searches and identification, screening, and eligibility of included studies.

A total of 2901 studies were initially identified. After removing duplicates, 2358 studies remained. These were screened by title and abstract, reducing the selection to 67 articles. Following a full-text analysis, 38 articles met the inclusion criteria (Figure 1). Most of the studies were published in English (*n* = 37, 97.36%), with one study published in Portuguese (*n* = 1, 2.64%). Analyzing the publications per year, the number of articles increased annually, with the highest number occurring in 2024 (*n* = 12), followed by 2023 (*n* = 9), 2022 (*n* = 9), 2021 (*n* = 7), and 2020 (*n* = 1).

Among the 38 articles included in the review, 26 (68.4%) were original research articles, six (1.5%) were short communications or brief reports, three (7.9%) were letters, two (5.3%) were conference papers, and one (2.6%) was a commentary. All articles were epidemiological studies, comprising 22 retrospective cohort studies, nine cross-sectional retrospective studies, five prospective surveillance studies, and two prospective cohort studies.

### 3.2. Viral Meningitis

#### 3.2.1. Enterovirus

Ten studies examined the epidemiology of enteroviral infections (EVs): four investigated enteroviral meningitis (EM); three analyzed meningitis in general and addressed the behavior of viral meningitis, including enteroviruses in their findings; one addressed general EVs in children; one pertained to general viral meningitis; and one explored the genetic diversity of echovirus 11 during the COVID-19 pandemic [8,9,10,11,12,13,14]. The countries studied were China, England, France, Germany, Israel, New Zealand, Poland, South Korea, Switzerland, and the United States of America (USA) [8,9,10,11,12,13,14].

Most studies reported a notable decrease in EM cases [8,9,10,11,12]. Five articles analyzed EM without focusing on specific enteroviral species [8,9,10,11,12]. The latest publications appeared in 2020 in the USA and 2021 in France and Switzerland, coinciding with the onset of the COVID-19 pandemic [8,9,10]. EM is seasonal in the northern hemispheres, encompassing the USA, France, and Switzerland, typically peaking in August, September, and October, with the lowest case numbers observed early in the year [8]. However, in 2020, the expected late summer/early fall EM peaks did not occur [8]. A study analyzing laboratory test positivity rates from 2018 to 2020 in the USA showed a sharp decline in EM cases. Among the 3914 tests performed, only 62 (1.6%) tested positive for enterovirus. In contrast, a significant peak (7.4%) was observed in September 2019. However, by September 2020, the positivity rate had dropped to 0% [8]. No EM cases were reported in other countries during the same period [9]. An 11-year retrospective study correlated EM trends with the introduction of various non-pharmaceutical interventions (NPIs) introduced to halt the COVID-19 pandemic in 2020. Stoffel et al. found no EM cases in 2020, despite a predictive count of 20 cases (95% CI, 12–29) for 2020 based on historical data in infants from 2010 to 2019 [9].

A retrospective study performed in France analyzed 4782 CSF samples from suspected community-acquired meningitis cases from January 2018 to August 2020 using the Biofire-FilmArray Meningitis/Encephalitis Panel; bioMérieux, Marcy-l’Étoile, France that performs the simultaneous detection of 14 pathogens, including enteroviruses [9]. The mean weekly percentage of CSF samples testing positive for EM was 0.2 ± 0.5% in 2020, which was significantly lower than the 16.0 ± 5.7% in 2018 and 10.0 ± 3.7% 2019 (*p* < 0.001) [10]. This was an important finding because the decrease in EM cases during 2020 was not linked to a reduction in CSF testing, as testing volumes were not affected by lockdown measures [10]. More recently, a prospective study by Kadambari et al. investigated the impact of the COVID-19 pandemic on EM cases using 22,114 laboratory-confirmed viral meningitis cases [11]. In 2013–2019, 15,299 cases (4.0/100,000, 95% confidence interval [CI]: 3.9–4.0) were reported, but the rate dropped markedly to 2061 cases (1.8/100,000, 95% CI: 1.7–1.9) in 2020–2021 [11].

A single-center study on EV at the Children’s Hospital, Zhejiang University School of Medicine, China, analyzed 34,152 samples collected between January 2019 and May 2023 from symptomatic children with a suspected enterovirus infection. Specimens included throat swabs, stool, pericardial fluid, ascitic fluid, sputum, and CSF. The study identified 1162 enterovirus-positive cases [12]. This study observed a marked decrease in cases in 2020; however, unlike other studies, enteroviral infections increased in 2021 and subsequent years [8,9,10,12]. The annual EV-positive rates ranged from 1.15% in 2020 to 5.46% in 2019 and from 4.43% in 2021 to 1.15% in 2020. After the COVID-19 pandemic, the EV rates decreased to 1.62% and 1.96% in 2022 and 2023, respectively [12].

Other studies have reported an increase in EM due to echovirus 6 [13,14]. One study noted an upsurge in EV outbreaks due to echovirus 6 in Israel in December 2021 before the emergence of the SARS-CoV-2 Omicron variant [13]. In January 2022, the number of EV cases decreased by 66%, coinciding with the peak of the Omicron wave [13]. However, by March 2022, a second increase in cases was observed [13]. Genomic sequencing confirmed the dominance of echovirus 6 (29%) before and after the Omicron wave [13]. Further phylogenetic analysis revealed that all 29 sequenced samples were similar and clustered with the echovirus 6 C1 subtype [13]. A similar trend was reported in Poland, where the researchers documented a rise in echovirus 11 circulation [14]. In contrast, although a new EV species and an intensification in the circulation of these agents were observed, the pandemic periods of 2020 and 2021 saw the lowest number of positive samples being collected, similar to previous studies [14]. Despite the lower number of positive samples during the pandemic between 2017 and 2023, Poland showed an increase from 2017 (*n* = 7) to 2023 (*n* = 124), representing 5.74% and 43.20% of the total number of isolates detected in the respective years [14]. Table 1 summarizes the EM studies performed during the COVID-19 pandemic period and enteroviral infection trends.

Three additional studies analyzed meningitis cases and noted reductions in meningitis cases, including EM, during the COVID-19 pandemic [15,16,17]. A multicenter study conducted in Germany observed a decline in viral meningitis, especially EM (*n* = 25 patients/year in 2020 vs. *n* = 97–181 patients/year between 2016 and 2019; all *p* < 0.001) [15]. Another longitudinal study from New Zealand (1991–2020) found a reduction in aseptic meningitis hospitalization rates in children aged >1 year in 2020, although rates had been increasing between 2004 to 2019 [15]. Most cases had unidentified pathogens (64%), but enteroviruses accounted for 29% of confirmed cases [16].

In South Korea, a retrospective study using electronic medical record data from five university hospitals identified 677 patients aged < 18 years with meningitis, although the causative pathogen was only confirmed in 137 patients [17]. The annual rate of the causative pathogens of meningitis before the COVID-19 outbreak showed that EV was the most common cause. The monthly incidence of EM further showed that there was seasonal variation, with a higher incidence during the summer. However, following the emergence of COVID-19, no cases of EM were reported, a trend attributed by the authors to the pandemic and associated public health restrictions [17].

#### 3.2.2. Other Viruses

Two studies have evaluated the impact of the COVID-19 pandemic on the circulation of other viruses. In a prospective study, Kadambari et al. found a decrease in other viruses, such as Human Parechovirus, during the pandemic. For example, a 64% decrease was observed in Human Parechovirus cases, with the incidence dropping from 0.1/100,000 in 2019 to 0.2/100,000 in 2021. However, after societal restrictions were lifted, Human Parechovirus levels rebounded to pre-pandemic levels [11].

Other viruses, such as cytomegalovirus, Epstein–Barr virus, and varicella zoster virus, have also been studied, but a minimal change was observed in their incidence during the pandemic [11]. A nationwide study from Japan evaluated the administrative medical payment system database and the diagnosis procedure combination, from 2016 to 2022. Among the 28,399 meningitis cases identified, 16,678 were identified as viral or aseptic in origin [18]. This study precisely documented a decrease in all meningitis cases during the COVID-19 pandemic, with significant reductions in cases of mumps meningitis in individuals aged 0 to 19 years and 20 to 29 years, in cases of herpes simplex virus (HSV) meningitis in the 20–49 age group, and cases of varicella zoster virus meningitis in individuals aged 20–49 years and 50 years [18].

#### 3.2.3. Aseptic Meningitis

A retrospective cross-sectional study analyzed the impact of the COVID-19 pandemic on aseptic meningitis in children in Iran between 2018 and 2022 [19]. Molecular methods to identify viruses have not yet been developed, although non-polio enteroviruses are considered the most important cause of aseptic meningitis in children in Iran. A total of 68 children were enrolled in the study, of whom 54 were admitted before the pandemic (54/13,286; 0.4%) and 14 after the pandemic (14/9406; 0.14%) [19]. Age-stratified analysis revealed a significant decrease in the incidence of aseptic meningitis in children older than 5 years after the COVID-19 outbreak (*p* < 0.001) [19]. Most patients were treated with acyclovir, though no cases of HSV-1 or HSV-2 were detected [19].

### 3.3. Bacterial Meningitis

Some studies have evaluated the incidence of bacterial meningitis, despite the variations in etiological agents [20,21,22,23]. A cross-sectional retrospective study in Brazil evaluated the epidemiology of hospital admissions for bacterial meningitis using the data from the Brazilian National Health System Database (Department of Informatics of the Public Unified Health System, DATASUS) for 2019 and 2020 [20]. There were 6921 hospitalizations recorded for bacterial meningitis, with 4091 (59.1%) occurring in 2019 and 2830 (40.9%) in 2020 [20]. Most hospitalized patients were males, of mixed ethnicity (brown), and <1-year old [20].

Brito et al. retrospectively evaluated notifiable diseases in Brazil, including meningitis, using the same centralized national database managed by DATASUS, focusing on the northern region. The results showed a decrease in the number of hospitalizations for meningitis in 2020 across all states compared with the 2015–2019 average, except for Amazonas and Rondônia, which experienced an increase in hospitalizations [21]. The states in the northern region with the largest reductions in meningitis-related hospitalizations were Amapá (−83%), Acre (−77%), and Pará (−70%) [21].

Jbari et al. observed a similar pattern of decrease in bacterial meningitis cases in central Morocco [22]. A retrospective epidemiological study was carried out in the Department of Pediatric Emergencies, Mother and Child Hospital, Marrakech, Morocco, focusing on meningitis cases during the lockdown period (March to May) in 2019 and 2020 [22]. The study identified 72 cases of suspected meningitis between March 2019 and March 2020, predominantly affecting boys (70%) and infants aged 1 month to 2 years (34%), similar to the findings of a Brazilian study [22]. The authors noted the impact of the COVID-19 pandemic on bacterial meningitis epidemiology. The final diagnosis of suspected meningitis was confirmed in 20% of the cases during the lockdown period compared to only 2.38% before the pandemic, and this difference was statistically significant (*p* < 0.05) [22]. The data from infectious disease notifications in the provincial territory of Messina, Italy, comparing the pre-pandemic (2017–2019) and the pandemic (2020–2022) periods, showed a 72% decrease in meningoencephalitis cases during the pandemic (*p* = 0.002) [23].

Specifically, bacterial meningitis cases decreased by 80.0% (*p* = 0.0028; OR = 0.2063) in the pandemic period compared to pre-pandemic, with meningococcal and pneumococcal meningitis cases decreasing by 80.0% and 87.5%, respectively (*p* = 0.0304; OR = 0.1146) [23]. Similarly, the data from Siracusa Local Health Authority, Italy, revealed a significant decline in vaccine-preventable invasive bacterial diseases, such as meningitis, with a decrease of 83.3% (*p* < 0.05) in the pandemic period (2020–2022) compared to pre-pandemic (2017–2019) levels [24].

#### 3.3.1. Meningococcal Disease

Six articles investigated the epidemiology of invasive meningococcal disease (IMD), one of which specifically focused on meningitis due to *Neisseria meningitidis* during the pandemic period [5,6,25,26,27,28].

A prospective surveillance study performed during the first 2 years of the COVID-19 pandemic in 30 countries provided the most robust data on invasive bacterial diseases through the Invasive Respiratory Infection Surveillance (IRIS) Consortium [6]. Overall, 116,841 cases were analyzed: 76,481 in 2018–2019 before the pandemic and 40,360 in 2020–2021 during the pandemic [25]. The IRIS consortium comprised 30 countries: Australia (*n* = 2184), Belgium (*n* = 4903), Brazil (*n* = 2378), Canada (*n* = 8707), China (*n* = 195), Hong Kong (*n* = 443), Colombia (*n* = 1515), Czech Republic (*n* = 1475), Denmark (*n* = 2166), England (*n* = 15,644), Finland (*n* = 2186), France (*n* = 3555), Germany (*n* = 10,293), Greece (*n* = 185), Iceland (*n* = 112), Ireland (*n* = 1177), Israel (*n* = 2074), Luxembourg (*n* = 127), New Zealand (*n* = 1792), Northern Ireland (*n* = 436), Paraguay (*n* = 617), Poland (*n* = 3396), Scotland (*n* = 1608), South Africa (*n* = 7394), South Korea (*n* = 106), Spain (*n* = 7063), Sweeden (*n* = 4115), Switzerland (*n* = 2917), the Netherlands (*n* = 2044), and Wales (*n* = 1159). Overall, the data on *N. meningitidis* revealed 2302 cases in 2018 (median: 192), followed by 2228 in 2019 (median: 182), and a marked reduction to 976 in 2020 (median: 40) [6]. When *N. meningitidis* cases were analyzed by serogroup and age, there was a significant reduction in infections across all serogroups, especially capsule groups W, C, and Y, with no changes in disease patterns by age group [6]. Moreover, the study reported a significant decrease in the incidence of invasive diseases caused by *N. meningitidis* and other bacteria, such as *S. pneumoniae* and *H. influenzae*, during the first 2 years of the pandemic, with cases beginning to increase in some countries toward the end of 2021 as pandemic restrictions were lifted [5,6].

A population-based study in Greece investigated the incidence of influenza and IMD in children aged 0–14 years during the COVID-19 pandemic [25]. The mean annual rate of IMD declined by 70%, with a mean annual incidence of 0.41 per 100,000 children in 2021–2021, compared to 1.38 per 100,000 in 2014–2019 [25].

Another study from France investigated the incidence of *H. influenzae* infection and IMD using the data from national databases before (2018–2019) and after (2020–2021) the COVID-19 pandemic in people at all ages [26]. The study reported 1595 IMD cases from 2017 to 2021, with the lowest case numbers of 202 and 106 in 2020 and 2021, respectively [26]. There were 791 serogroup B cases (49.6%), 286 serogroup C cases (17.9%), 271 serogroup W cases (17%), 221 serogroup Y cases (13.9%), and 26 from other groups and non-groupable cases (1.6%). The yearly distribution of the serogroups over the studied 5 years showed a decreasing number of cases since the declaration of the COVID-19 pandemic compared to the period prior to this pandemic, although the decrease seemed to be less prominent in 2021 than in 2020 [26]. Interestingly, serogroup C presented an early decrease in 2018, which continued until 2020 and 2021 [26].

The decrease in IMD documented in France was later confirmed by another retrospective analysis based on the data from the French National Reference Centre for Meningococci and *H. influenzae* [27]. However, by 2022, a rebound in IMD incidence was observed. Notably, serogroups W and Y were significantly associated with IMD cases that evolved to sepsis (*p* < 0.0001). The proportion of meningeal presentations was significantly higher before the COVID-19 pandemic, decreasing from 44.1% (240 cases of 544 clinical cases in 2015) to 32.7% (156 cases of 477 clinical cases in 2022) [27]. An important finding of this study was the increase in general IMD after the pandemic, as well as the increase in atypical forms, such as meningococcal pneumonia and bacteremic abdominal forms, which were linked to higher mortality rates post-COVID-19 [27]. A study utilizing Taiwan’s National Notifiable Disease Surveillance System database (2019–2020) documented a reduction in meningococcal meningitis and invasive pneumococcal diseases. The decrease in meningococcal meningitis was 16.7% in 2020 compared with 2019 [28]. The same study found that no imported meningococcal cases were reported in either 2019 or 2020 [28].

#### 3.3.2. *Streptococcus pneumoniae*

The most common streptococcal species that cause meningitis are *S. pneumoniae* and *S. agalactiae* [1,2]. Two studies specifically investigated the incidence of pneumococcal meningitis during the COVID-19 pandemic, whereas three others analyzed trends in invasive bacterial infections, including *S. pneumoniae*, over the same period [5,6,27,28,29,30,31].

The IRIS consortium documented *S. pneumoniae* infections between 2018 and 2020, reporting 62,837 cases between 1 January 2018 and 31 May 2020 (week 22 of 2020) [5,6]. Their model estimated a 38% decrease in the incidence of reported invasive *S. pneumoniae* infections (0.62; 95% CI: 0.55–0.70) followed by an additional 13% average weekly reduction up to the end of the study period (May 31, 2020; 0.87; 95% CI: 0.84–0.89) [5]. When the year dataset was expanded to include 2021, there were significant reductions in all major serotypes of *S. pneumoniae* in 2020–2021, although cases due to some serotypes, mainly 19A, 4, 7C,15BC, 23 B, 23F, 35F, and 10B, began to increase in late 2021 [6].

A secondary database study from the USA investigated changes in the incidence of bacterial diseases caused by *S. pneumoniae*, *H. influenzae*, and other streptococci between the pandemic period from 1 March to 31 December 2020 [29]. The observed incidence of *S. pneumoniae* infections was 58% lower than the expected incidence, making it the most significantly reduced bacterial infection, followed by *H. influenzae*, which declined by 60% [29].

Studies conducted in Uruguay and the Netherlands reported comparable declines [30,31]. In Uruguay, a national database study registered a decline in pneumococcal meningitis in the early pandemic period (2020–2021), April 2020, and October 2021 (0.17; 95% CI: 0.03–0.3). This difference was statistically significant when compared with the incidence before COVID-19 (2017–2019) (0.57; 95% CI: 0.3–0.7; *p* = 0.005) and post-COVID-19 (2022) (0.83; 95% CI: 0.3–0.82; *p* = 0.0006) [30]. A prospective surveillance study in the Netherlands analyzed 1210 cases recorded by the Netherlands Reference Laboratory for Bacterial Meningitis between 2014 and 2023. The typical seasonal increase in pneumococcal meningitis in September–March was absent from 2020 to 2021. Conversely, a smaller peak was observed in summer 2020. Further analysis revealed a 50% decrease in cases by 2022, with numbers returning to pre-pandemic levels by 2023 [31].

Taiwan, through its national database system, reported a 45.2% reduction in invasive pneumococcal disease from 2019 to 2020 (with cases dropping from 352 to 193), and a 100% decrease in imported cases during the same period (from two to 0 cases) [28].

#### 3.3.3. *Streptococcus agalactiae*

*S. agalactiae*, also known as Lancefield Group B Streptococcus (GBS), is a well-known meningitis pathogen in infants less than 90 days [1,2]. Four studies were included in this analysis, all of which examined general invasive bacterial infections, including *S. agalactiae* [5,6,27,32]. Global data revealed that *S. agalactiae* infections did not decrease in 2018–2021 unlike other bacterial pathogens, such as *N. meningitidis* and *S. pneumoniae* [5,6]. Samples positive for *S. agalactiae* from nine IRIS laboratories in 2020 were included to determine whether the decrease in other pathogens was due to disruptions in sample submission. Only the data from the northern hemisphere were collected [6]. No difference in sample submission was observed during this period, confirming that the number of *S. agalactiae* infections did not decrease compared to pre-pandemic levels (1.02; 95% CI: 0.75–1.40) [5,6]. A population-based surveillance study from the USA detected a decline in GBS infections, similar to what was observed for *S. pneumoniae*, *N. meningitidis*, and *H. influenzae*, during the early pandemic period (March 2020) [29]. When analyzed broadly, compared with the expected incidences based on mathematical models, the GBS was 12% lower [29]. However, the same study noted that, although the overall decline in incidence was sustained throughout the rest of 2020 for other bacteria, this was not the case for GBS [29].

A large multicenter cross-sectional study involving 97 hospitals in the USA and Canada examined the prevalence of urinary tract and invasive bacterial infections in febrile infants during the COVID-19 pandemic, covering the period from January 2020 to November 2022 [32]. Among the 9112 patients included, 43 (0.5%) were diagnosed with bacterial meningitis, with GBS being the most common cause (44.2%), followed by *E. coli* (30.2%) [32]. An analysis of the trends in meningitis cases revealed a monthly decline in the age-adjusted odds of an infant developing meningitis (OR, 0.91; 95% CI: 0.86–0.97). The highest prevalence of bacterial meningitis was recorded in February 2021 (1.7%) and decreased to 0% by March 2022 [32].

#### 3.3.4. *Streptococcus pyogenes*

*Streptococcus pyogenes*, also called Lancefield Group A Streptococcus (GAS), is a ubiquitous Gram-positive coccus that colonizes the human skin and nasopharynx. While GAS commonly causes pharyngitis and upper respiratory infections, it rarely leads to meningitis [31,32,33,34,35]. This analysis included 10 articles focused on the changes in the epidemiology of streptococcal infections, five of which focused specifically on invasive GAS infections. Two of these studies emphasized post-pandemic trends [33,34].

An increase in GAS infections was noted in the Netherlands between July 2021 and June 2022, compared to pre-pandemic levels [33,34]. A survey conducted across seven hospitals recorded 61 cases between 1 July 2021 and 30 June 2022, compared to 56 cases from January 2018 to December 2019. Most cases occurred during the first 3 months of 2022 or the post-pandemic period, with a peak of 28 cases in the second quarter, representing a 3-fold and 14-fold increase compared to 2018–2019 [33].

A separate study from the Netherlands conducted by the National Invasive Group A streptococcal infection notification using the data from the Dutch National Reference Laboratory for Bacterial Meningitis confirmed this trend. The study revealed a seven-fold increase in the number of notifiable invasive GAS infections among children aged 0–5 years compared with pre-pandemic years [34]. Among 42 cases in this age group, seven had preceding or coinciding varicella zoster infections, and nine were fatal [34]. In both Dutch studies, the most common diagnoses were pneumonia with empyema and pediatric necrotizing fasciitis, a clinical condition not observed in the pre-COVID-19 period in the second study [33,34].

In the USA, a single-center study in Texas analyzed GAS infections in infants less than 1-year of age from 2012 to 2022 and reported a different pattern from that observed in the Netherlands [35]. While invasive GAS cases increased through 2019, the incidence decreased during the first 2 years of the COVID-19 pandemic in this population. However, a resurgence was noted post-COVID-19, in the second quarter of 2022 [35]. Similar to a Dutch study, GAS predominantly caused pneumonia/pleural empyema (*n* = 7; 21.8%). Other reported infections included deep neck abscesses (*n* = 4; 12.5%), bacteremia without focus (*n* = 4; 12.5%), osteomyelitis (*n* = 3; 9.4%), retropharyngeal/parapharyngeal abscesses, and meningitis (*n* = 2, 6.3%) [35]. During the study period, 504 pediatric GAS cases were recorded, with 32 cases in infants (6.3%); eight of which (25%) occurred in the final quarter of 2022 [34,35].

In the post-COVID-19 era, the incidence of GAS infections was reported worldwide [36,37]. An Italian survey analyzed 2580 children from January and December 2023, finding that 20.3% had GAS infections [36]. The most common symptoms included sore throat (76.9%), fever (75.2%), tonsillar exudates (25.2%), lymphadenopathy (21.8%), and scarlet fever (14.7%). One patient was hospitalized for GAS meningitis [36]. The authors hypothesized that GAS cases decreased during COVID-19, followed by a significant resurgence afterward [36]. A retrospective study in Poland covering the COVID-19 analyzed 45 children with a median age of 7 years, 31 (69%) of whom had invasive GAS infections.

The diagnoses were sepsis with toxic shock syndrome (13%), deep soft tissue infections (10%), meningitis (6%), pneumonia (6%), or respiratory tract infections (12%). Mania et al. stated that previous Polish data showed an incidence rate of 8.2/100.00 in 2020 and 5.7/100.00 in 2021, but once pandemic restrictions were lifted, case numbers increased significantly in 2022 and 2023 [37].

#### 3.3.5. *Haemophilus influenzae*

Five articles examined *H. influenzae* infections during the COVID-19 pandemic [5,6,28,29,38] The IRIS consortium initially reported data from 26 countries between January 2018 and May 2020, of which 24 submitted data on *H. influenzae* (for a total of 7796 cases) [5]. The data were obtained from Belgium, Brazil, China, the Czech Republic, Denmark, England, Finland, France, Germany, Hong Kong, Iceland, Ireland, Israel, Luxembourg, the Netherlands, New Zealand, Northern Ireland, Poland, Scotland, South Africa, South Korea, Sweden, Switzerland, and Wales [5]. A substantial and sustained reduction in the number of invasive cases of *H. influenzae* infection diagnosed between March and May 2020 was observed compared to the pre-pandemic period [5].

A subsequent larger study from the IRIS consortium analyzing the data from 30 countries, including 2021, performed a meta-analysis and calculated the risk of invasive disease during the pandemic for each bacterial species by hemisphere [6]. *H. influenzae* presented a decreased risk (0.51; 95% CI: 0.40–0.66) in both northern and southern hemispheres [6]. In addition, the stratification of *H. influenzae* cases by serotype showed a reduction in case counts of all serotypes except serotype b, which decreased in 2020 and then increased by the end of 2021 (*p* < 0.0001) [6]. Serotypes f, a, e, non-b, d, and c decreased annually from 2018 to 2021. When analyzed by country, the rise in *H. influenzae* type b (Hib) infections in 2021 was most notable in five nations: the Netherlands (*n* = 70), France (*n* = 50), South Africa (*n* = 43), Israel (*n* = 27), and Paraguay (*n* = 10). It is noteworthy that despite this increase in Hib cases in 2021, the numbers remained lower compared to pre-pandemic levels [6].

In Taiwan, a retrospective study evaluating airborne/droplet-transmitted infectious diseases between 2019 and 2020 found an increase in three diseases during the pandemic (2020): legionellosis (227 cases in 2019 vs. 241 cases in 2020), invasive Hib infection (one case in 2019 vs. three cases in 2020), and hantavirus syndrome (one case in 2019 vs. 10 cases in 2020) [28].

Although the percentage increase in Hib cases was 200%, the absolute case count remained low, and only serotype B was analyzed [28]. In the USA, *H. influenzae* infections decreased by 60% in 2020, immediately after the pre-pandemic period [29]. This decrease was followed by the implementation of COVID-19 control measures [29]. Stratified analyses revealed significantly lower than expected incidences for *S. pneumoniae* and *H. influenzae* across all age groups. The observed invasive *H. influenzae* incidence rate ratio per 100,000 inhabitants (0.7) was lower overall and by age, race, and surveillance site, compared to the expected rate (1.7) from March to December 2020, reflecting a substantial decline (0.4; 95% CI: 0.33–0.5) [29]. This study analyzed all *H. influenzae* serotypes.

Other countries have also reported an increase in the incidence of Hib infections. In the Netherlands, Hib incidence increased by 40% in 2020 compared to 2019 mainly in non-vaccinated individuals from the Netherlands [38]. No changes were observed in serotypes e and f; however, an overall increase was noted in *H. influenzae* in 2020 [38]. In the Netherlands, Hib incidence was particularly elevated in clustered religious municipalities within the Bible Belt, where vaccine coverage is substantially lower. However, a higher incidence (1.45/100,000) was also observed outside the Bible Belt, with incidence rates of 0.35 and 0.32 per 100,000 in 2020 and 2021, respectively, compared to 0.17 and 0.27 per 100,000 in 2015–2019 [38].

#### 3.3.6. *Listeria monocytogenes*

The data on invasive *Listeria monocytogenes* infections and meningitis are scarce. However, a large nationwide study in Spain described the trends in invasive *L. monocytogenes* infections from 2000 to 2021 [39]. Using the data from the National Registry of Hospital Discharges, the study identified 8192 hospital admissions due to *L. monocytogenes* [39]. Among these cases, the most common clinical presentations were meningoencephalitis (*n* = 3112, 38.2%), sepsis (*n* = 962, 11.8%), endocarditis (*n* = 77, 0.9%), and neonatal listeriosis (*n* = 450, 5.5%). During the study period, the overall incidence of *L. monocytogenes* infections increased, from five per one million population in 2000 to 8.9 in 2021 (*p* < 0.001). A sharp increase in cases was observed in 2019, primarily due to a large foodborne disease outbreak in Andalusia. However, when solely analyzing the COVID-19 pandemic peaks in 2020, after a significant increase in admissions during 2019 (13.5 per one million population), there was a drastic decrease in 2020 (7.2 per one million population) [39].

#### 3.3.7. Other Causative Pathogens and Meningitis Due to Drug-Resistant Bacteria

Some studies have addressed *E. coli* invasive infections, a possible cause of meningitis, mainly in infants younger than 90 days [1,2]. Two studies specifically addressed *E. coli* meningitis, one focusing on community-acquired infections and another on antibiotic-resistant strains [32,40].

A large multicenter cross-sectional study involving 97 hospitals across the USA and Canada found a downward trend in bacterial meningitis cases in children, with 33% of the cases caused by *E. coli* [32]. A multicenter retrospective study performed by the Shandong Province Pediatric Antimicrobial Resistance Surveillance System network evaluated drug-resistant bacterial meningitis by testing positive CSF cultures from 2017 to 2022 [40]. A total of 5793 CSF isolates were collected with the predominant pathogens, including coagulase-negative Staphylococcus (CoNS), *S. pneumoniae*, and *E. coli* in children, and the top three pathogens were CoNS, *Acinetobacter baumannii*, and *Klebsiella pneumoniae* in adults. After the pandemic, an increase was observed in the abundance of *E. coli* (*p* = 0.0067), *K. pneumoniae*, *Pseudomonas aeruginosa*, *Enterobacter cloacae*, and *Enterococcus faecalis* (*p* < 0.05).

In contrast, the prevalence of CoNS (*p* = 0.0039) and *A. baumannii* (*p* = 0.0059) decreased significantly [40]. Beyond the changes in bacterial incidence, this study also documented shifts in antimicrobial resistance patterns. After COVID-19, the resistance rates of CoNS to erythromycin, SXT, quinupristin/dalfopristin (Q/D), and tetracycline decreased (*p* < 0.05). *A. baumannii* showed significant differences in resistance rates to cefoperazone/sulbactam, ampicillin/sulbactam, ceftriaxone, ceftazidime, cefepime, and imipenem before, during, and after the pandemic (*p* < 0.05) [40]. *K. pneumoniae* showed no differences in resistance rates during the study period [40]. Table 2 provides a summary of studies revealing the major bacterial agents causing meningitis and their trends during the COVID-19 pandemic. Table 3 summarizes the major etiological meningitis agents, modes of transmission, and factors associated with the epidemiological changes during the COVID-19 pandemic.

## 4. Discussion

The COVID-19 pandemic is a significant public health challenge worldwide, influencing the epidemiology of meningitis by altering the circulation of viral, bacterial, and even multidrug-resistant microorganisms [23,24,25,26,27,28,29,30,31,32,33,34,35,36,37,38,39,40]. Our study highlights an increase in publications aimed at understanding the impact of COVID-19 on other diseases, such as meningitis, with most studies published between 2023 and 2024 [11,12,14,18,22,23,24,25,26,27,28,29,30,31,32,33,34,35,36,37,38,39,40]. With the re-establishment of healthcare services and the apparent return to post-pandemic normalcy, the resurgence of meningitis cases, the impacts observed in 2020–2021 are becoming increasingly evident and have been the focus of more in-depth studies [4,5,6,7,8,9,10,11,12,13,14,15,16,17,18,19,20,21,22,23,24,25,26,27,28,29,30,31,32,33,34,35,36,37,38,39,40,41].

The COVID-19 pandemic has altered the incidence and distribution of viral and bacterial meningitis, potentially owing to changes in healthcare access, viral coinfections, and shifts in public health priorities. Specifically, the widespread implementation of COVID-19 prevention measures, such as social distancing and mask-wearing, may have reduced the transmission of certain pathogens that cause meningitis. Simultaneously, COVID-19 or its associated complications may increase the risk of meningitis in specific populations.

Nevertheless, many studies attributed the changes to the COVID-19 pandemic but did not provide specific causative measures in their country or context to better justify the trends [19,23,24]. It is likely that mask usage contributes to a reduction in the transmission of droplet-borne pathogens. However, in the case of enteroviruses, social distancing measures and the closure of schools, daycare centers, and other services may have played a significant role in this reduction [8,9,10,11,12,13,14,15,16,17]. Most articles have attributed the observed changes to the impact of COVID-19; however, some studies have also reported a decline in vaccination coverage, which may have influenced disease patterns in the years following the pandemic.

Enteroviruses are among the most common viruses infecting humans worldwide [1,2]. More than 100 types of bacteria are known to infect humans, most of which are frequently detected globally [8,9,10,11,12,13,14,15,16,17]. The impact of the COVID-19 pandemic on EM and other severe enteroviral infections is well-documented [8,9,10,11,12,13,14,15,16,17]. Enterovirus transmission occurs predominantly via the fecal–oral route through person-to-person contact, requiring human interaction, and affecting adults and children, who are the most affected [11,12,13,14]. Other forms of EV transmission rarely occur via droplets [11]. To understand how SARS-CoV-2 influences the EV infection rate, it is essential to understand their transmission routes [8,9,10,11,12,13,14,15,16,17].

For an EV to occur, the introduction of infected stool or mucus into the oral cavity of an uninfected person is required. The most likely cause of the decrease in EV cases was the implementation of public health measures implemented to reduce SARS-CoV-2 transmission, such as social distancing and lockdowns [8,9,10,11,12,13,14,15,16,17]. Another possible explanation is viral interference between enteroviruses and SARS-CoV-2. Because enteroviruses are shed in respiratory secretions and stools, person-to-person transmission remains the primary mode of transmission. This hypothesis is consistent with reports of other respiratory viruses that have experienced attenuation during the pandemic [4,5,6,7].

At the beginning of the pandemic, there were concerns about the potential increase in secondary bacterial diseases associated with prior COVID-19 infections (similar to what was observed with outbreaks of influenza and respiratory syncytial viruses) [4]. However, these concerns were unfounded. In contrast, a decline was observed in most bacterial infections caused by *S. pneumoniae*, *N. meningitidis*, and *Haemophilus influenzae* [5,6]. However, a resurgence occurred between late 2021 and early 2022, depending on the region and the characteristics of the local strains [5,6]. Two large-scale studies comprehensively documented the decline in major meningitis pathogens during the COVID-19 pandemic across 30 countries in both hemispheres [5,6].

In 2024, a narrative review analyzed the IMD-causing serogroups epidemiology changes before and after the COVID-19 pandemic [42]. Successful vaccination programs must be tailored to local epidemiology, which varies geographically, temporally, by age, and by serogroup, particularly before the COVID-19 pandemic [42]. The COVID-19 pandemic has changed the epidemiology of all IMD serogroups worldwide and has been followed by a resurgence of serogroups that were prevalent locally before the pandemic, affecting unvaccinated age groups. Counterintuitively, IMD rates had been declining in many parts of the world before the COVID-19 pandemic, largely because of the implementation of meningococcal vaccination programs in regions such as the Americas and Europe [42].

In Spain, only one case of IMD was recorded by week 31 of 2021, compared to 24 cases in 2018 and 21 cases in 2019 [42]. Similarly, in Poland, where the MenC vaccination is recommended for individuals aged 2 months to 19 years, 62 cases were reported by week 39 of 2021, a significant decrease from the 116 and 121 cases reported in 2018 and 2019, respectively [42]. In South America, Brazil and Chile reported substantial reductions in confirmed IMD cases in 2020, with decreases of 65.0% and 91.3%, respectively, compared with 2019 [42].

Other reviews have broadly addressed IMD, highlighting the rebound observed after the easing of pandemic peaks [43]. Countries such as the United Kingdom, the United States, and England experienced a significant increase in IMD cases toward the end of 2021 and beginning of 2022, following the relaxation of lockdown measures [43].

France presented a reduction in IMD incidence in 2020–2021, presented an increase in 2022 above pre-pandemic levels [43]. A systematic review focused on meningitis in the Niger Republic during and after the COVID-19 pandemic and emphasized the unique challenges and patterns observed in this context [43]. Most meningitis cases in Niger are caused by *N. meningitidis* serogroups C1 and W [43]. A total of 231 cases of meningitis were reported from 1 November 2021 to 31 January 2022. Recently, 559 cases of meningitis, including 18 deaths, occurred in the Zinder region, southeast of the Niger Republic, from 1 November 2022 to 27 January 2023 [43].

An increase in meningococcal outbreaks in the Niger Republic region was noted after the COVID-19 pandemic, which was attributed to the spatial clustering of cases and variations in climatic factors, suggesting the role of the environment and transmission in driving epidemics [43].

In addition, the absence of significant changes in *S. agalactiae* infections is an important finding [5,6,27,30]. Thus, this trend has not drawn much attention, as *S. agalactiae* is not usually transmitted through droplets [27,30]. Most of the data collected were from the northern hemisphere, but it is likely that the southern hemisphere exhibits a similar pattern, given the seasonal and epidemiological similarities of this pathogen between the two hemispheres [5,6].

Similar to other bacterial pathogens, a notable increase in invasive GAS infections was observed post-pandemic [33,34,35,36]. Several hypotheses have been proposed to explain this rise, including the relaxation of control measures such as social distancing, mask usage, and lockdowns. Although large-scale studies have documented a decline in the number of invasive GAS infections during the pandemic, few have specifically evaluated GAS meningitis [33,34,35,36]. This may be due to the predominance of more common etiological agents in meningitis cases, such as *S. pneumoniae*, *N. meningitidis*, and *H. influenzae*.

Our study did not establish a trend in invasive *L. monocytogenes* infection. The only available study found a decreasing trend in invasive *L. monocytogenes* infections, which may be related to the healthcare measures taken to control the COVID-19 pandemic [38]. Although a nationwide study was included, no other studies on invasive *L. monocytogenes* infections were identified. The challenges associated with this issue may be attributed to the rarity of listeriosis and the absence of specific data from national databases worldwide [39]. Our study had some limitations. A meta-analysis could not be conducted because of the heterogeneity of the included articles, which varied in methodologies, approaches, and age groups. Additionally, uncommon and rare causative agents of meningitis were not analyzed because most reports consisted of single cases and were therefore excluded from this study. Furthermore, the risk of bias in the included studies was not assessed because of the diversity of the study designs.

This study provides an overview of the trends and epidemiology of meningitis and causative pathogens during and post-COVID-19 pandemic. We believe that other viral and bacterial causative agents of meningitis may also reduce COVID-19 measures. Further research on the impact of COVID-19 on other infectious diseases is necessary to better understand the potential of pandemics to affect the environment and public health. To the best of our knowledge, this is the first review to prioritize understanding the impact of the pandemic on the circulation of meningitis-causing agents rather than respiratory agents.

## 5. Conclusions

Herein, we present a systematic review of the changes and trends in the epidemiology of major etiological agents of meningitis during and after the COVID-19 pandemic. Epidemiological patterns varied by country, pathogen, and patient age. Despite heterogeneity among studies, the findings suggest that the COVID-19 pandemic may have influenced the epidemiology of multiple microorganisms both during and after the pandemic. Understanding these shifts in meningitis etiological agents is crucial for guiding control measures, vaccination strategies, and public health policies. However, the data from developing countries remain limited. Further research on various meningitis pathogens is essential to gain a more comprehensive understanding of COVID-19’s global impact on infectious disease transmission. More studies are needed to better understand the role of COVID-19 in these dynamic changes.

## Figures and Tables

**Table 1:** Summary of epidemiological studies assessing the influence of COVID-19 pandemic measures on enterovirus infections by country.

Country	Study Period	Number	Summary of Data	*p*-Value	Trends of Cases
USA [8]	2012–2020	3129	92.6% reduction in positivity rates in 2020	N/A	Decrease
Switzerland [9]	2010–2020	288	100% reduction in EV meningitis cases in 2020 (0 cases)	N/A	Decrease
France [10]	2018–2020	15	98.4% reduction in EV meningitis cases in 2020	*p* < 0.001	Decrease
German [15]	2016–2020	540	78.4% decline in EV meningitis cases in 2020 compared to 2019	*p* < 0.001	Decrease
New Zealand [16]	1991–2020	5142	52.2% reduction in aseptic meningitis in the median hospitalization rate in 2020 *	N/A	Decrease
Korea [17]	2017–2020	116	100% reduction in EV meningitis cases in 2020 (0 cases)	*p* < 0.001	Decrease
Israel [13]	2021–2022	98	66% reduction in EV meningitis in January 2022 and 78% increase in March 2022	N/A	Decrease in January 2022 and increase in March 2022
England [11]	2013–2023	13,585	77.7% significant decline in EV meningitis cases during 2020–2021 ^1^	N/A	Decrease
China [12]	2019–2023	1162	21.1% decline in 2020; 385% and 82.6% increase in 2021 and 2023.	*p* < 0.0001	Decrease in 2020 and 2023; increase in 2021
Poland [14]	2016–2023	266 ^2^ 11 ^3^	100% decline in 2020 and 2021	N/A	Decrease in 2019–2020 and increase in 2022–2023

Legend: N/A: not available; EV: enterovirus. * EV was the most prevalent etiological agent identified (29%) ^1^ and disruption of seasonal trends of EV, with peaks in winter months. ^2^ Isolates. ^3^ Cases.

**Table 2:** Summary of studies assessing the influence of COVID-19 pandemic measures on bacterial etiological agents of meningitis.

Country	Study Period	Etiological Agents	Number of Cases	Summary of Data	*p*-Value	Trends
^1^ 26 countries [5]	2018–2020	*S. pneumoniae*, *H. influenzae*, *N. meningitidis*, *S. agalactiae*	7651	A significant and sustained reduction in invasive diseases due to *S. pneumoniae*, *H. influenzae*, and *N. meningitidis* in early 2020. No significant changes in the incidence of invasive *S. agalactiae* infections were observed	*p* < 0.0001	Decrease
^2^ 30 countries [6]	2018–2021	*S. pneumoniae*, *H. influenzae*, *N. meningitidis*, *S. agalactiae*	116,841	Significant reductions were observed in: *S. pneumoniae* (49.6%), *H. influenzae* (41.2%), and *N. meningitidis* (66.3%). In contrast, *S. agalactiae* showed a slight increase (5.7%) in average of cases	*p* < 0.0001	Decrease and increase *
Italy [23]	2017–2022	*S. pneumoniae*, *N. meningitidis*	32	80% decrease in bacterial meningoencephalitis. Meningococcal and pneumococcal forms decreased by 80% and 87.5%	*p* < 0.0001	Decrease
Italy [24]	2017–2022	All bacteria	N/A	Vaccine-preventable invasive bacterial diseases decreased by 83.3% in the post-pandemic period	*p* < 0.05	Decrease
Italy [36]	2023 (January–May)	*S. pyogenes*	358	20.3% GAS infection post-COVID-19. A single patient was hospitalized with GAS meningitis	N/A	Not informed
Brazil [20]	2019–2020	All bacteria	6921	Bacterial meningitis decreased 30.1% between 2019 and 2020	N/A	Decrease
Brazil [21]	2015–2020	All bacteria	N/A	Meningitis decreased 73% between 2019 and 2020	N/A	Decrease
Morocco [22]	2019–2020	All bacteria	72	Bacterial meningitis was 6 times more frequent during containment	*p* < 0.05	Increase
Greece [25]	2014–2021	*N. meningitidis*	N/A	70% decrease in the mean annual rate of IMD in 2020–2021	N/A	Decrease
France [26]	2017–2021	*H. influenzae*, *N. meningitidis*	1595 ^3^ 808 ^4^	IMD cases decreased by 52.9% and 75.2% in 2020 and 2021. IHiD cases decreased by 21.2% in 2020 and increased by 3.1% in 2021	*p* < 0.0001	Decrease
France [27]	2015–2022	*N. meningitidis*	2719	IMD decreased by 47.7% and 72.8% in 2020 and 2021. Meningococcal meningitis decreased from 44.1% to 32.7% in seven years	*p* = 0.03	Decrease
Taiwan [28]	2019–2020	*S. pneumoniae*, *H. influenzae*, *N. meningitidis*	545 ^5^ 11 ^6^ 4 ^4^	IPD and meningococcal meningitis decreased 45.2% and 16.7% during the pandemic. IHiD increased from 1 to 3 cases in the same period	N/A	Decrease
Uruguay [30]	2017–2022	*S. pneumoniae*	100	Pneumococcal meningitis decreased by 79.7% by the late-pandemic phase (2022; 29 cases)	*p* = 0.005	Decrease
USA [29]	2014–2020	*S. pneumoniae*, *H. influenzae*, *S. agalactiae*, *S. pyogenes*	1,019,887	Incidences of *S. pneumoniae*, *H. influenzae*, GAS, and GBS were 58%, 60%, 28%, and 12% lower during the pandemic period of 2020, respectively	N/A	Decrease
USA/Canada [32]	2020–2022	*E. coli*, *S. agalactiae*	9112	Monthly downward trend in the incidence of bacterial meningitis due *S. agalactiae* (44.2%) and *E. coli* (30.2%)	N/A	Decrease
USA [35]	2012–2018	*S. pyogenes*	504	83.3% decrease in iGAS cases between 2019 and 2020. In 2021 and 2022, there were, respectively, 0 and 10 cases	N/A	Decrease
Netherlands [31]	2014–2023	*S. pneumoniae*	1210	Between 2014 and 2023, 1210 cases of pneumococcal meningitis were identified, with an IR of 1.02 (2014–2020). During the pandemic (2020–2022), IR dropped by approximately 50%	N/A	Decrease
Netherlands [33]	2021–2022	*S. pyogenes*	117	121.7% (5.1/month) increase in pediatric iGAS cases compared to the pre-pandemic period (2.3/month)	N/A	Increase
Netherlands [34]	2016–2021	*S. pyogenes*	319	118% increase in non-puerperal iGAS cases compared to the pre-pandemic period	N/A	Increase
Netherlands [38]	1992–2021	*H. influenzae*	N/A	Increase in the overall incidences in 2020 (0.39 per 100,000) and 2021 (0.33 per 100,000). It remained below 0.3 cases per 100,000 from 1996 to 2019	N/A	Increase
Poland [37]	2022–2023	*S. pyogenes*	45	69% presenting iGAS, meningitis detected in 2 cases	N/A	Not informed
Spain [39]	2000–2021	*L. monocytogenes*	8152	50% decrease in hospitalizations in 2020 compared to 2019. Meningoencephalitis corresponds to 38.2% of cases	N/A	Decrease
China [40]	2017–2023	CoNS, *S. pneumoniae*, *E. coli*, *S. aureus*, *S. agalactiae*, *E. faecium*, *A. baumannii*, *E. faecalis*, *H. influenzae*, *K. pneumoniae*	5793	26.5% and 13.1% reduction in infections in the pediatric and adult population between 2019 and 2020	*p* = 0.0039	Decrease

Legend: * *Haemophilus influenzae* B; IMD: invasive meningococcal disease; IPD: invasive pneumococcal disease; IHiD: invasive *Haemophilus influenzae* disease; GAS: Group A Streptococcus; GBS: Group B Streptococcus; iGAS: Invasive Group A Streptococcus; CoNS: Coagulase-negative Staphylococci. ^1^ Belgium, Brazil, Canada, China, Czech Republic, Denmark, England, Finland, France, Germany, Hong Kong, Iceland, Ireland, Israel, Luxembourg, the Netherlands, New Zealand, Northern Ireland, Poland, Scotland, South Africa, South Korea, Spain, Sweden, Switzerland, and Wales. ^2^ Australia, Belgium, Brazil, Canada, China, Colombia, Czech Republic, Denmark, England, Finland, France, Germany, Hong Kong, Greece, Iceland, Ireland, Israel, Luxembourg, the Netherlands, New Zealand, Northern Ireland, Paraguay, Poland, Scotland, South Africa, South Korea, Spain, Sweden, Switzerland, and Wales. ^3^ IMD. ^4^ IHiD. ^5^ IPD. ^6^ Meningococcal meningitis.

**Table 3:** Pathogens, transmission route, and factors associated with changes in transmission.

Etiological Agent	Transmission Route	Hypotheses
*Neisseria meningitidis* [5,6,25,26,27,28]	Droplets	Decrease in transmission due to public health measures; disruption on routine invasive disease surveillance.
*Haemophilus influenzae* [5,6,28,29,38]	Droplets	Decrease in transmission due to public health measures; potential reduction in viral co-infections that facilitate bacterial infection; failure in Hib vaccination in 2020 and 2021.
*Streptococcus pneumoniae* [5,6,27,28,29,30,31]	Droplets	Decrease in transmission due to public health measures.
*Streptococcus agalactiae* [5,6,27,32]	Droplets and contact	No significant change observed.
*Streptococcus pyogenes* [31,32,33,34,35]	Droplets and contact	Decrease in transmission due to public health measures.
*Escherichia coli* [32,40]	Fecal–oral, healthcare-associated	Antibiotic use changes, immune system modulation, or altered hospital transmission.
Listeria monocytogenes [39]	vertical transmission, contaminated food, contact	Decrease in transmission due to public health measures.
Enterovirus [8,9,10,11,12,13,14]	Fecal–oral. Rarely droplets	Decrease in transmission due to public health measures.
HSV-1 e HSV-2 [18]	Contact ^1^	Not directly affected by COVID-19, but stress and immunosuppression could influence reactivation rates.

Legend: HSV: herpes simplex virus; *Haemophilus influenzae* b. ^1^ and sexual transmission.

## Data Availability

Dataset available on request from the authors.

## Supplementary Materials

The following supporting information can be downloaded at: https://www.mdpi.com/article/10.3390/tropicalmed10030081/s1.
