# Supplementary material for: Alterations and Dynamics of Major Meningitis Etiological Agents During and Post-COVID-19 Pandemic: A Systematic Review
Source: Trop Med Infect Dis. 2025 Mar 18;10(3):81. doi: 10.3390/tropicalmed10030081 (PMC11945666; doi:10.3390/tropicalmed10030081)
Supplement: Supplementary file 1 [file tropicalmed-10-00081-s001.zip › tropicalmed-3534389-supplementary.pdf]

NIHR | National Institute for Health and Care Research

PROSPERO
International prospective register of systematic reviews

Alterations and Dynamics of Major Meningitis Etiological Agents During and Post-COVID-19 Pandemic: A Systematic Review

Luís Arthur Brasil Gadelha Farias, Lourrany Borges Costa

Citation
Luís Arthur Brasil Gadelha Farias, Lourrany Borges Costa. Alterations and Dynamics of Major Meningitis Etiological Agents During and Post-COVID-19 Pandemic: A Systematic Review. PROSPERO 2025 CRD420250643445. Available from https://www.crd.york.ac.uk/PROSPERO/view/CRD420250643445.

REVIEW TITLE AND BASIC DETAILS

Review title

Alterations and Dynamics of Major Meningitis Etiological Agents During and Post-COVID-19 Pandemic: A Systematic Review

Original language title

Alterations and Dynamics of Major Meningitis Etiological Agents During and Post-COVID-19 Pandemic: A Systematic Review

Review objectives

Has the COVID-19 pandemic influ- 70

enced the global circulation of meningitis etiological agents?

Keywords

Covid-19; Enterovirus; Haemophilus influenzae; Meningitis; Neisseria meningitidis; Pandemic; Streptococcus pneumoniae

SEARCHING AND SCREENING

Searches

PubMed (MEDLINE); Embase; SciELO; LILACS.

Study design

Both randomized and nonrandomized study types will be included.

Included

We searched for randomized and non-randomized controlled study designs, nonrandomized controlled trials, cohort studies, case-control studies, cross-sectional studies, outbreak reports, genomic studies, systematic literature reviews if a meta-analysis was included.

Excluded

Case reports and series were excluded. A case series was defined as one comprising 20 or fewer patients and characterized 87

by the uncontrolled nature of these studies

Link to search strategy

A full search strategy is not available.

ELIGIBILITY CRITERIA

Condition or domain being studied

Etiological agents of meningitis during COVID-19 pandemic

Population

Included

We only included and thoroughly analyzed articles in English, Spanish and Portuguese. We also reviewed the retrieved articles' references to uncover additional studies the initial search strategy might have missed.Studies were included in the review if they reported the following primary outcomes:

(1) meningitis epidemiological data such as incidence, prevalence, or serotype distribution during or post-COVID-19; (2) etiological agents of meningitis, including invasive/atypical disease presentations to better describe trends in pathogen circulation during and post-COVID-19; (3) comparative data on meningitis etiological agents before, during, and after the COVID-19 pandemic. Additionally, studies that described the etiological agents of meningitis across all age groups were considered.

Intervention(s) or exposure(s)

Included

Articles contemplating meningitis data during a period before the COVID-19 pandemic were only included when comparing data during or after the COVID-19 pandemic. Articles with emphasis on etiological agents of meningitis and invasive disease presentations were also included to better describe the trends of circulation of these agents during pandemic.

Comparator(s) or control(s)

Included

None.

Context

We employed a rigorous methodology to minimize potential biases. Two independent reviewers assessed the titles and abstracts of the articles to select those that met the predefined inclusion criteria. We resolved any discrepancies by consensus or, if needed, by consulting a third reviewer. Two infectious diseases specialists and one epidemiologist with experience in infectious diseases epidemiology reviewed the articles. We designed this process to reduce selection bias and ensure a comprehensive and unbiased literature representation.

OUTCOMES TO BE ANALYSED

Main outcomes

Studies were included in the review if they reported the following primary outcomes: (1) Articles contemplating meningitis epidemiological data, such as prevalence, incidence, and seroprevalence, or serotypes distribution, during or Post-COVID-19 pandemic. (2) Articles with emphasis on etiological agents of meningitis epidemiology and invasive disease presentations were also included to better describe the trends of circulation of these agents during or Post-COVID-19 pandemic. (3) Data on etiological agents before COVID-19 pandemic were included when comparing data during or after the COVID-19 pandemic. We considered studies describing etiologic agents of meningitis in all age groups.

Measures of effect

Prevalence, incidence, and seroprevalence, or serotypes distribution; confidence intervals and significance whem comparing pré and post-COVID-19 pandemic.

Additional outcomes

None

DATA COLLECTION PROCESS

Data extraction (selection and coding)

The search period will be from January 2020 to December 2024, and included the following terms to retrieve relevant

articles: "Meningitis" OR "Etiological agents of meningitis" OR "Streptococcus pneumoniae" OR "Enterovirus" OR "Neisseria meningitidis" OR "Haemophilus influenzae" OR "Strepto coccus agalactiae" OR "Listeria monocytogenes" AND "COVID-19 Pandemic" OR "COVID-19" OR "New coronavirus" OR "Pandemic.

We will included articles in English, Spanish, or Portuguese.

We will also review the reference lists of retrieved articles to identify additional relevant stud ies that might have been missed in the initial search strategy. We included randomized and non-randomized controlled studies, non-randomized controlled trials, cohort studies, case control studies, cross-sectional studies, outbreak reports, genomic studies, and systematic literature reviews that included a meta-analysis. However, case reports and series were ex cluded. A case series was defined as one comprising 20 or fewer patients and characterized

by the uncontrolled nature of these studies[7]. Mendeley (https://www.mendeley.com), free reference management software, was used to organize references, manage duplicates, and streamline article selection. Full text articles identified during the screening process were retrieved for detailed analysis.

Studies will be include in the review if they reported the following primary outcomes: (1) meningitis epidemiological data such as incidence, prevalence, or serotype distribution during or post-COVID-19; (2) etiological agents of meningitis, including invasive/atypical disease presentations to better describe trends in pathogen circulation during and post-COVID-19; (3) comparative data on meningitis etiological agents before, during, and after the COVID-19 pandemic. Additionally, studies that describes the etiological agents of meningitis across all age groups will be considered.

Two independent reviewers will assess the titles and abstracts of articles to select those that met the predefined inclusion criteria. Any discrepancies were resolved by consensus or, if needed, by consulting a third reviewer. Two infectious disease specialists and an epidemiologist with experience in infectious disease epidemiology reviewed the articles. We designed this process to reduce selection bias and ensure comprehensive literature representation.

Risk of bias (quality) assessment

We employed a rigorous methodology to minimize potential biases. Two independent reviewers assessed the titles and abstracts of the articles to select those that met the predefined inclusion criteria. We resolved any discrepancies by consensus or, if needed, by consulting a third reviewer. Two infectious diseases specialists and one epidemiologist with experience in infectious diseases epidemiology reviewed the articles. We designed this process to reduce selection bias and ensure a comprehensive and unbiased literature representation.

PLANNED DATA SYNTHESIS

Strategy for data synthesis

Data will be categorize by etiological agent whenever available and were further classified into bacterial or viral meningitis when the specific pathogen was not identified or specified in the article. For this review, the pre-pandemic period was defined as 2019, the pandemic period spanned from January 2020 to December 2021, and the post-pandemic period encompassed 2022 and subsequent years.

Mendeley (https://www.mendeley.com), a free reference management software, will be used to organize references, manage duplicates, and streamline article selection.

REVIEW AFFILIATION, FUNDING AND PEER REVIEW

Review team members

Dr Luís Arthur Brasil Gadelha Farias (review guarantor). Hospital São José de Doenças Infecciosas (HSJ). Brazil.

No conflict of interest declared.

Professor Lourrany Borges Costa. Universidade de Fortaleza (Unifor). Brazil.

No conflict of interest declared.

Named contact

Professor Lourrany Borges Costa (lourranybc@unifor.br). Universidade de Fortaleza (Unifor). Brazil.

Review affiliation

Hospital São José de Doenças Infecciosas (HSJ)

Funding source

Additional non-commercial funding information
None

TIMELINE OF THE REVIEW

Review timeline

Start date: 25 January 2025. End date: 12 March 2025.

Date of first submission to PROSPERO

03 March 2025

Date of registration in PROSPERO

03 March 2025

CURRENT REVIEW STAGE

Publication of review results

Results of the review will be published in English.

Stage of the review at this submission

Review stage	Started	Completed
Pilot work		
Formal searching/study identification		
Screening search results against inclusion criteria		
Data extraction or receipt of IPD		
Risk of bias/quality assessment		
Data synthesis		

Review status

The review is currently planned or ongoing.

ADDITIONAL INFORMATION

Additional information
This review will me part of the doctorate program for one of the authors.

PROSPERO version history

- Version 1.0, published 03 Mar 2025

Review conflict of interest
Declared individual interests are recorded under team member details.. No additional interests are recorded for this review.

Country
Brazil

Other registration details
None

Medical Subject Headings
COVID-19; Humans; Meningitis; Pandemics

Details of any existing review of the same topic by the same authors
None

Disclaimer
The content of this record displays the information provided by the review team. PROSPERO does not peer review registration records or endorse their content.

PROSPERO accepts and posts the information provided in good faith; responsibility for record content rests with the review team. The guarantor for this record has affirmed that the information provided is truthful and that they understand that deliberate provision of inaccurate information may be construed as scientific misconduct.

PROSPERO does not accept any liability for the content provided in this record or for its use. Readers use the information provided in this record at their own risk.

Any enquiries about the record should be referred to the named review contact
